# HPLC Evaluation of Phenolic Profile, Nutritive Content, and Antioxidant Capacity of Extracts Obtained from *Punica granatum* Fruit Peel

**DOI:** 10.1155/2013/296236

**Published:** 2013-08-01

**Authors:** Sushil Kumar Middha, Talambedu Usha, Veena Pande

**Affiliations:** ^1^Department of Biotechnology, Bhimtal Campus, Kumaun University, Nainital, Uttarakhand 263136, India; ^2^Department of Biotechnology & Biochemistry, Maharani Lakshmi Ammanni College for Women, Malleswaram, Bangalore, Karnataka 560012, India

## Abstract

This study revealed polyphenolic content, nutritive content, antioxidant activity, and phenolic profile of methanol and aqueous extracts of *Punica granatum* peel extract. For this, extracts were screened for possible antioxidant activities by free radical scavenging activity (DPPH), hydrogen peroxide scavenging activity and ferric-reducing antioxidant power (FRAP) assays. The total phenolics and flavonoid recovered by methanolic (MPE) and the water extract (AQPE) were ranged from 185 ± 12.45 to 298.00 ± 24.86 mg GAE (gallic acid equivalents)/gm and 23.05 ± 1.54 to 49.8 ± 2.14 quercetin (QE) mg/g, respectively. The EC_50_ of herbal extracts ranged from 100 *µ*g/ml (0.38 quercetin equivalents), for AQPE, 168 *µ*g/ml (0.80 quercetin equivalents), for MPE. The phenolic profile in the methanolic extracts was investigated by chromatographic (HPLC) method. About 5 different flavonoids, phenolic acids, and their derivatives including quercetin (1), rutin (2), gallic acid (3), ellagic acid (4), and punicalagin as a major ellagitannin (5) have been identified. Among both extracts, methanolic extract was the most effective. This report may be the first to show nutritive content and correlation analysis to suggest that phenols and flavonoids might contribute the high antioxidant activity of this fruit peel and establish it as a valuable natural antioxidant source applicable in the health food industry.

## 1. Introduction

Nature still obliges as the man's primary source for the cure of his ailments. Research in preventive medicine showed the importance of functional nutrition in reducing the risk factor of certain chronic diseases. Innate defense system of the human body may be insufficient for the damage caused by continued oxidative stress [1]. Reactive oxygen species (ROS) are, in general, considered to be cytotoxic and are implicated in the progression of cancer, inflammation, radiation injury, and aging [2]. Growing scientific evidence has shown adverse side effects, like liver damage and mutagenesis, of synthetic antioxidants [3]. Therefore, recently there has been an upsurge of interest in natural products as antioxidants, as they inhibit the free radical reactions and protect human body from various diseases, such as cancer and diabetes [4]. Recent studies showed that a number of plant products including polyphenolic substances (e.g., gallocatechins, delphinidin, cyanidin, gallic acid, ellagic acid, pelargonidin and sitosterol) and various plants or herbal extracts exert potent antioxidant actions, which are very well known for their healing powers [5].

The pomegranate, *Punica granatum Linn*. (*Punicaceae *family), is natively from the Himalayas in northern India to Iran, nevertheless it has been cultivated and naturalized since ancient times over the entire Mediterranean region. Over the past decade, significant progress has been made in establishing the pharmacological mechanisms of different parts of pomegranate and the individual constituents responsible for them [6–9]. Previous *in vivo* and *in vitro* studies on fruit peel illustrated its role in facilitating the reversal of glucose level in diabetic rats [7, 8]. However, to date, no studies regarding the correlation between antioxidant activity and total phenols of *P. granatum* peels (PGP) have been conducted. Therefore, the perception of this study is to evaluate the antioxidant activity by measuring DPPH scavenging activity, reducing power, flavonoid content of methanol, and aqueous extract of PGP. The study also demonstrates the correlation between antioxidant activity and total phenols using HPLC. 

## 2. Materials and Methods

### 2.1. Collection and Authentication of Plant Material

*Punica granatum* was purchased from local market of Bhowali, Bhimtal (Uttarakhand) during the month of July and were identified, authenticated, and deposited (KU/D007) to Botany Department Herbarium, Kumaun University, Nainital, Uttarakhand. Plant material was shade-dried at room temperature. 

### 2.2. Preparations of Aqueous and Methanolic Extract

Aqueous extract of PGP (AQPE) was prepared by traditional method [3]. Powdered material of PGP (11 kg) was successively extracted with methanol (1 : 10 ratio) using a soxhlet apparatus [7]. The extracts were stored at 4°C until required. Before use, the methanolic PGP extract (MPE) was dissolved in double-distilled water (DDW) in desired concentrations and was used as test substances. Total yield of AQPE and MPE were 6.8% and 4.8%, respectively. 

### 2.3. Preliminary Phytochemical Screening

The presence or absence of the phytochemical constituents in both the extracts were analyzed using standard procedures for carbohydrates, reducing sugars, tannins, saponins, flavonoids, steroids, alkaloids, anthraquinones, glycosides as prescribed by Goyal et al., 2010 [10].

### 2.4. Assessment of Antioxidant Activity

#### 2.4.1. DPPH Radical Scavenging Assay

The free radical scavenging capacity of the AQPE and MPE against DPPH was determined spectrophotometrically by the modified method of Goyal et al., 2010 [10]. % scavenging of the DPPH free radical was measured using the following equation: % DPPH radical-scavenging = [(absorbance of control − absorbance of test Sample)/(absorbance of control)] × 100.

#### 2.4.2. Assay of Reducing Power

Reducing power assay was also carried out as described previously by Goyal et al., 2010 [10]. The absorbance of the final reaction mixture of two parallel experiments was expressed as mean ± standard deviation. Augmented absorbance of the mixture indicates stronger reducing influence of the extract.

#### 2.4.3. Scavenging of Hydrogen Peroxide

The capability of the extracts to forage hydrogen peroxide was determined according to Goyal et al., 2010 [10].

### 2.5. Quantitative Analysis of Antioxidant Compounds

#### 2.5.1. Determination of Total Phenols, Flavonoids, and Proanthocyanidin

Total phenolic content, flavonoids, and proanthocyanidin were determined by procedures that were adopted in author's previous studies [10]. 

#### 2.5.2. High-Performance Liquid Chromatography Analysis (HPLC)

The HPLC system (Waters, Singapore) consisted of photodiode array detector (W2998), dual pump system (515 waters), temperature control module II (TC2 waters), pump control module (PC2 waters), system controller (EMOAA01712), and a reverse phase HPLC analytical column waters Spherisorb C8, 4.6 × 100 mm, 5 *μ*m particle size. The flow rate was adjusted to 1.2 mL/min; the detector was set at 220, 240, 260, 270, and 280 nm at 1.2 nm resolution with the mobile phase 0.1% methanol : phosphoric acid (50 : 50 v/v, isocratic mode). Active constituent of MPE extract was dissolved in a mixture of methanol and water (6 : 4 v/v) and identified by comparison of the retention time in chromatogram with standard gallic acid, rutin, quercetin, ellagic acid, and punicalagin (Sigma Chemical Co, St. Louis, USA). Data analysis was done using Empower software.

### 2.6. Physicochemical Characterization

Analyses of reducing and total sugars, total moisture content, protein, saponification value, iodine number, fructose, coumarins and crude fibers were carried out using the methods defined by AOAC [11] in triplicates.

## 3. Result and Discussion

### 3.1. Phytochemical Analysis

The phytochemical analysis conducted on PGP extract revealed the presence of saponins, steroids, alkaloids, tannins, carbohydrates, flavonoids, flavonols, anthraquinone, proanthocyanidins, glycosides, reducing sugars, and total phenols. These phytochemical compounds are known to support bioactive activities in medicinal plants [12] and thus responsible for the antioxidant activities of this plant extract used in the study.

### 3.2. Phenolic and Flavonoid Concentration

In the last decade there are numerous publications proving the antioxidant activity of many plant extract, due to the presence of the phenolic compounds [13]. However the results are incomparable since they were tested through various methods. Therefore the results of our research shows that values of total phenolic compounds in AQPE and MPE are 185 ± 2.45 GAE mg/gm dry weight and 298 ± 4.86 GAE mg/gm dry weight, respectively (Table 1).

Besides any other secondary metabolites, mostly plants show their antioxidant activities due to phenylpropanoid derivatives, such as polyphenols. The antioxidant activities of PGP may also be due to its polyphenol content [14]. Phenolic compounds are the prime antioxidant components of natural products and are composed of phenolic acids and flavonoids, which are potent radical terminators. They donate an electron to radicals and break the reaction of lipid oxidation at the initiation step [15, 16].

The total flavonoid and flavonol contents of the AQPE and MPE were 23.05 ± 1.54 and 49.8 ± 2.14 quercetin equivalent/g and 0.39 ± 0.04 and 0.44 ± 0.032, respectively, with reference to standard curve (absorbance = 0.001 quercetin (*μ*g) + 0.0238 (*R* ^2^ = 0.9965) (Table 1).

### 3.3. Reducing Power Assay

**Figure 1:**
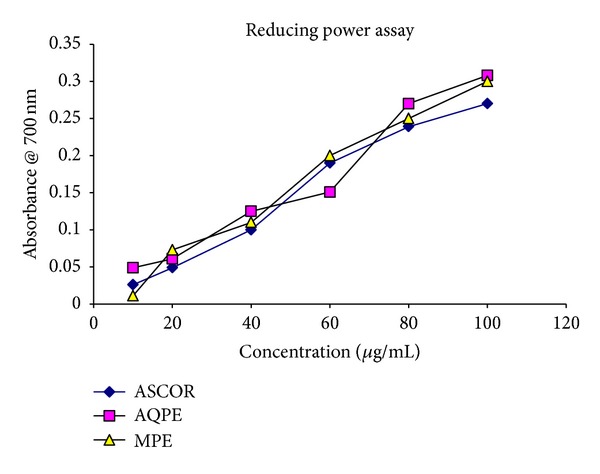
The effect of different concentrations of *P. granatum* peel extracts and ascorbic acid (ASCOR) reducing power assay. Values are means ± SE of analyses done in triplicate. *Concentration of extracts in increasing order: 10, 20, 40, 60, 80, and 100 *μ*g/mL; concentration of ascorbic acid in increasing order: 0.005, 0.01, 0.02, 0.03, 0.04, and 0.05 *μ*g/mL.

The reductive capabilities of the plant extract compared with ascorbic acid have been depicted in Figure 1. The reducing power of the extract of AQPE and MPE were found to be notable, which increased gradually with a rise in the concentration. As illustrated in Figure 1, Fe^3+^ was transformed to Fe^2+^ in the presence of the extract and the reference compound ascorbic acid. At 100 *μ*g/mL, the absorbance of the AQPE, MPE, and ascorbic acid was 0.30, 0.26, and 0.28, respectively, while at 80 *μ*g/mL, the absorbance of both the extract and ascorbic acid was almost the same. From Figure 1, it can be inferred that AQPE exhibits maximum reducing capability in comparison with MPE and the standard ascorbic acid.

Ferric-reducing antioxidant power (FRAP) measures the ability of antioxidants to reduce ferric 2, 4, 6-triperidyl-s-triazine complex to intensively blue colored ferrous complex in acidic medium. Hence any compound which has redox potential lower than that of redox pair Fe^3+^/Fe^2+^ can theoretically reduce Fe^3+^ to Fe^2+^ [15]. The activity of these reductones is to break radical chain by donation of a hydrogen atom, indicating that antioxidative properties are related to the increase in reducing power [7, 10]. Therefore, the marked antioxidant properties in different extracts may be related to its higher reducing power.

### 3.4. Hydrogen Peroxide Scavenging

**Figure 2:**
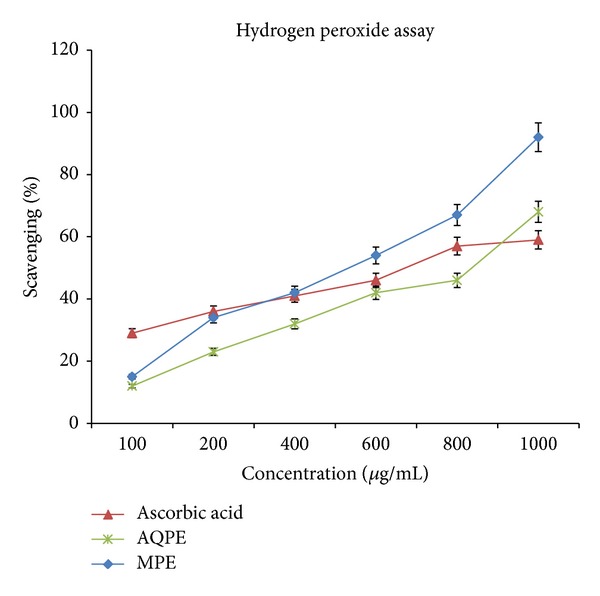
Hydrogen peroxide scavenging of the aqueous and methanolic extract of *P. granatum* in comparison with a standard (ascorbic acid) at *λ* = 230 nm.

Figure 2 shows that the MPE and AQPE extracts are good scavenger of H_2_O_2_ (IC_50_ = 426.74 ± 52.61 mg/mL and 478.8 ± 12.61 mg/mL) compared with standard ascorbic acid (IC_50_ = 678 ± 0.3 mg/mL). The IC_50_ value of the extract was lesser than that of the standard.

### 3.5. DPPH Scavenging Activity

**Figure 3:**
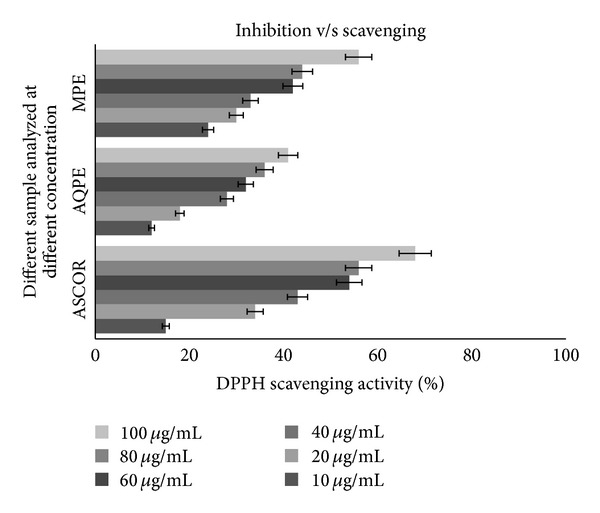
Dose inhibition curve of the aqueous and methanolic extract of *P. granatum *peel on DPPH scavenging activity. Values are means ± SE of analyses done in triplicate. *Concentration of hydrolyzed extracts in increasing order: 10, 20, 40, 60, 80 and 100 *μ*g/mL; concentration of ascorbic acid in increasing order: 0.005, 0.01, 0.02, and 0.03 *μ*g/mL.

The results of the DPPH scavenging activity of PGP are shown in Figure 3. The scavenging ability of MPE and AQPE were comparable to ascorbic acid.

IC_50_ values of AQPE and MPE were noted to be 100 *μ*g/mL and 168 *μ*g/mL, respectively. Prior reports have also indicated the same kinds of the effects [16, 17]. DPPH accepts an electron or hydrogen radical to become a diamagnetic molecule. As the electron becomes paired of in the presence of free-radical scavenger the color absorption vanishes and the resulting decoloration stoichiometrically coincides with the number of electrons taken up. Hence the bleaching of DPPH absorption is a representative of the capacity of the test materials. 

*In situ*, free radicals like polyaromatic hydrocarbon cations have been linked with carcinogenesis [18]. Thus, products that will scavenge DPPH *in vitro* may also scavenge poly-aromatic hydrocarbon cations *in vivo*. DPPH radical scavenging activity of MPE was significantly higher (*P* > 0.05) than AQPE at lower concentrations (10, 20, and 40 *μ*g/mL).

This study specified the MPE extract's higher phenolic ability and antioxidant activity than AQPE. Our findings are in agreement with prior studies [7, 9, 17]. Methanol is efficient solvent due to its less polar nature than water; hence it helps in releasing polyphenols from the plant cell easily. The decrease in antioxidant activity of AQPE was in agreement with the amount of phenol quantity. 

### 3.6. Nutrient Content of Pomegranate Peel

PGP showed high nutritive value and contains important raw materials like crude fibers, protein, and carbohydrates. Composition of MPE exhibited the moisture content (5.40–18.135%), iodine number (233.49), saponification number (1.122), reducing sugars (16.94), proteins (4.90), and fructose (15.622). 

**Figure 4:**
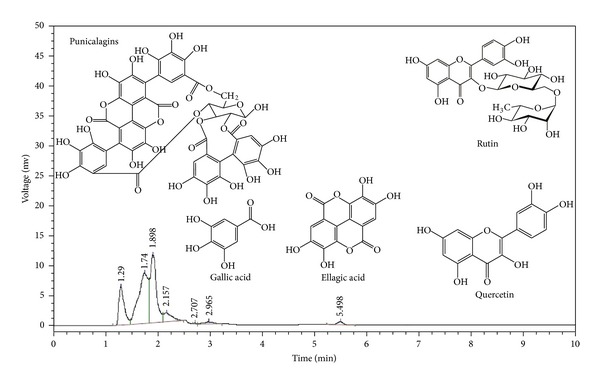
The *Punica* peels chromatograms gallic acid, rutin, quercetin, ellagic acid, and punicalagin, respectively.

In order to investigate components from the MPE, the HPLC analysis was performed (Figure 4). This HPLC analysis revealed the presence of some major phenolic compounds such as gallic acid and ellagic acids in addition to punicalagin as a major ellagitannin (Figure 4). All these compounds are deduced to be the major components from the experimental results and from the literature reported previously [7]. Authors' findings are in accordance with previous reports of HPLC analysis of MPE, detected gallic acid (34.03%), and catechin (3.31%) [18]. Ben Nasr et al., 1996 [19] have also reported that pomegranate peel contains ellagic acid, ellagitannins, and gallic acids.

### 3.7. Correlation Studies

Results obtained in the antioxidant assays were well correlated with total phenol and total flavonoids in both MPE and AQPE extracts see Supplementary Materials available online at http://dx.doi.org/10.1155/2013/296236 (S I, S II, S III). The total phenol was significantly positively correlated (*R* ^2^(*M*) = 0.852, *R* ^2^(*A*) = 0.781) with DPPH, respectively, whereas total flavonoids were positively correlated (*R* ^2^(*M*) = 0.710, *R* ^2^(*A*) = 0.330) with DPPH in both the extracts. A better linear relationship exists between hydrogen peroxide scavenging and total phenols in MPE as compared to AQPE. The reducing power of both the extracts was also positively correlated with hydrogen peroxide scavenging (*R* ^2^(*M*) = 0.573, *R* ^2^(*A*) = 0.686) and positively correlated with total flavonoids (*R* ^2^(*M*) = 0.337, *R* ^2^(*A*) = 0.609) and total phenols (*R* ^2^(*M*) = 0.530, *R* ^2^(*A*) = 0.274). These results are in agreement with prior studies reported [3, 20].

## 4. Conclusion

The data presented here indicates that the marked antioxidant activity of MPE, that contains large amounts of flavonoids and phenolic compounds, may act in a similar fashion as reductones by donating the electrons and reacting with free radicals to convert them into more stable product and terminate free radical chain reaction. These *in vitro* assays indicate that this PGP extract is a significant source of natural antioxidants. On the other hand, the unknown minor components present have not been elucidated in terms of their activity. It is timeconsuming to purify all antioxidants, one by one, from the PGP. From the practical point of view, a suitable extracting procedure should be developed to recover as many antioxidants as possible before an extract rich in natural antioxidants could be further explored for possible applications in health-promoting supplements for the clinical use and in food industry. 

## Supplementary Material

S I: Nutrient content of MPE per 100 g.
S II: Linear correlation coefficient of *Punica granatum* peel methanolic extract.
S III: Linear correlation coefficient of *Punica granatum* peel aqueous extract.

## Figures and Tables

**Table 1:** Preliminary phytochemical screening of the *Punica granatum peel* extract.

Chemical compounds	Result	AQPE	MPE
Saponins	+	QND	QND
Steroids	+	QND	QND
Alkaloids	+	QND	QND
Tannins	+	QND	QND
Carbohydrates	+	QND	QND
Flavonoids^a^	+	23.05 ± 1.54	49.8 ± 2.14*
Flavonols^a^	+	0.39 ± 0.04	0.44 ± 1.54 *µ*g
Anthraquinone	+	QND	QND
Proanthocyanidins^b^	+	9.09 ± 0.86	14.09 ± 1.56
Glycosides	+	QND	QND
Reducing sugars	+	QND	16.94 ± 0.39 mg/100 mg
Total phenol^c^	+	185 ± 12.45	298 ± 24.86*
Coumarins	+	QND	QND

QND: quantity not determined, −: compound not detected, +: compound detected, whereas AQPE, aqueous, and MPE, indicate methanolic extract.^a^Total flavonoids and flavonols content analyzed as quercetin equivalent (QE) mg/g of extract; values are the average of triplicates. ^b^Total proanthocyanidins content analyzed as catechin acid equivalent (GAE) mg/g of extract; values are the average of triplicates. ^c^Total phenol content analyzed as gallic acid equivalent (GAE) mg/g of extract; values are the average of triplicates. *Significant difference between aqueous and methanolic extract at *P* < 0.05.
