# Supplementary material for: HPLC Evaluation of Phenolic Profile, Nutritive Content, and Antioxidant Capacity of Extracts Obtained from Punica granatum Fruit Peel
Source: Adv Pharmacol Sci. 2013 Aug 1;2013:296236. doi: 10.1155/2013/296236 (PMC3747345; doi:10.1155/2013/296236)
Supplement: Supplementary file 1 — S I: Nutrient content of MPE per 100 g. S II: Linear correlation coefficient of Punica granatum peel methanolic extract. S III: Linear correlation coefficient of Punica granatum peel aqueous extract. [file 296236.f1.docx]

| **Composition** | **Content** |
| --- | --- |
| Total Solid | 94.50 |
| Moisture | 5.40-18.135% |
| Total Sugars | 17.70 |
| Reducing Sugars | 16.94 |
| Protein | 7.90 |
| Iodine Number | 233.496g |
| Saponification value | 1.122mg |
| Fructose | 15.622 ± 2.81 mg/100mg |

S I: Nutrient content of MPE per 100g

S II : Linear correlation coefficient of *Punica granatum* peel methanolic extract

| AQPE | **DPPH** | **FRP** | **H_2_O_2_** | **TP** | **TF** |
| --- | --- | --- | --- | --- | --- |
| **DPPH** | 1 |  |  |  |  |
| **FRP** | 0.5884 | 1 |  |  |  |
| **H_2_O_2_** | 0.6381 | 0.6860 | 1 |  |  |
| **TP** | 0.7813 | 0.6747 | 0.2741 | 1 |  |
| **TF** | 0.33051 | 0.6090 | 0.6069 | 0.78803 | 1 |

S III : Linear correlation coefficient of *Punica granatum* peel aqueous extract

|  | MPE | **DPPH** | **FRP** | **H_2_O_2_** | **TP** | **TF** |
| --- | --- | --- | --- | --- | --- | --- |
|  | **DPPH** | 1 |  |  |  |  |
|  | **FRP** | 0.9719 | 1 |  |  |  |
|  | **H_2_O_2_** | 0.9883 | 0.5739 | 1 |  |  |
|  | **TP** | 0.8522 | 0.4288 | 0.5301 | 1 |  |
|  | **TF** | 0.7104 | 0.3377 | 0.3924 | 0.9463 | 1 |
